# A Rare Case of Catamenial Pneumothorax and a Review of the Current Literature

**DOI:** 10.7759/cureus.42006

**Published:** 2023-07-17

**Authors:** Krupa K Solanki, Micah Shook, Jojo Yorke, Amanda Vanlandingham

**Affiliations:** 1 Pulmonology and Critical Care, East Tennessee State University - Quillen College of Medicine, Johnson, USA; 2 Internal Medicine, Norton Community Hospital, Norton, USA; 3 Pulmonology, Johnson City Medical Center, Johnson, USA

**Keywords:** catamenial pneumothorax, video-assisted thorascopic surgery, gnrh analogues, awareness about contraceptives, pulmonary bleb wedge resection, pleurodesis, thoracic endometriosis, contraceptive, thoracostomy tube, vats

## Abstract

A 34-year-old female smoker, with a history of pelvic endometriosis, presented with initial symptoms of shortness of breath and a choking sensation. She was found to have a right pneumothorax on chest x-ray. Over the next eight months, she ultimately underwent three tube thoracostomies, two video-assisted thoracoscopic surgeries (VATS), wedge resection, and repeated pleurodesis due to pneumothorax recurrence. She was seen multiple times post-surgically with the focus of treatment being smoking cessation rather than contraceptive therapy, despite an early follow-up visit noting that the initial symptoms coincided with her menstruation.

The purpose of this article is to bring attention to this rarely diagnosed condition. With added awareness and understanding of the underlying causes and available treatments, medical providers could likely spare many women from similar experiences and dramatically improve the quality of their lives.

## Introduction

A catamenial pneumothorax (CP) is a rare and poorly understood condition that affects young and middle-aged women. It is characterized by recurrent pneumothoraces that occur within 72 hours before or after the start of menstruation [1]. It is one presentation of thoracic endometriosis syndrome (TES) and can present with chest pain, hemoptysis, and hemothorax [2,3]. CP is underdiagnosed in women presenting with initial spontaneous pneumothoraces (SP) [4,5], despite estimations that they account for up to 2-6% of all SP [1] and up to 33% of SP in women [4]. Here, we present a case of a late-diagnosed CP to illustrate a need for awareness of this condition and review current treatment modalities.

## Case presentation

**Figure 1:**
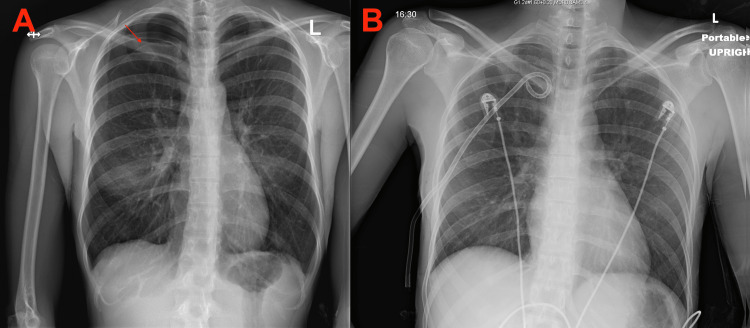
Chest x-ray A showed right-sided pneumothorax measuring approximately 3.2 cm from the chest wall. Chest x-ray B showed minimal apical pneumothorax after thoracostomy tube placement.

A 34-year-old female smoker (0.5 packs per day) with a history of anxiety presented to the hospital with shortness of breath and a choking sensation. She had been seen by her primary care physician a few days prior for nausea, vomiting, diarrhea, and abdominal pain and had been treated for viral gastroenteritis. An initial physical exam revealed decreased breath sounds on the right. She was found to have right-sided pneumothorax on chest x-ray (CXR) (Figure 1A) and underwent small-bore thoracostomy tube placement (Figure 1B) with eventual pneumothorax resolution and tube removal. Despite an early follow-up visit noting that her initial symptoms coincided with her menstruation, she was not offered contraceptive therapy.

**Figure 2:**
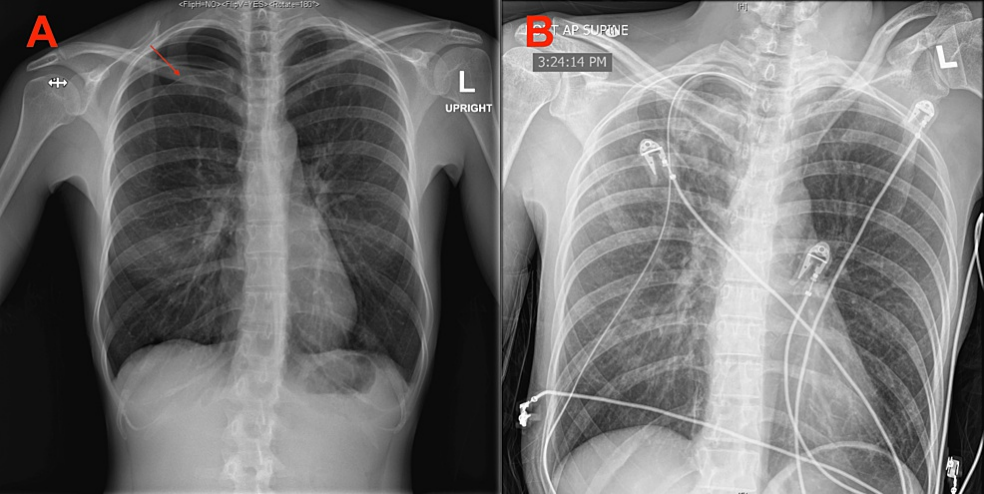
Chest x-ray A showed a recurrent right-sided pneumothorax that measured 3.7 cm from the apex. Chest x-ray B showed the resolution of the pneumothorax following thoracostomy tube placement.

**Figure 3:**
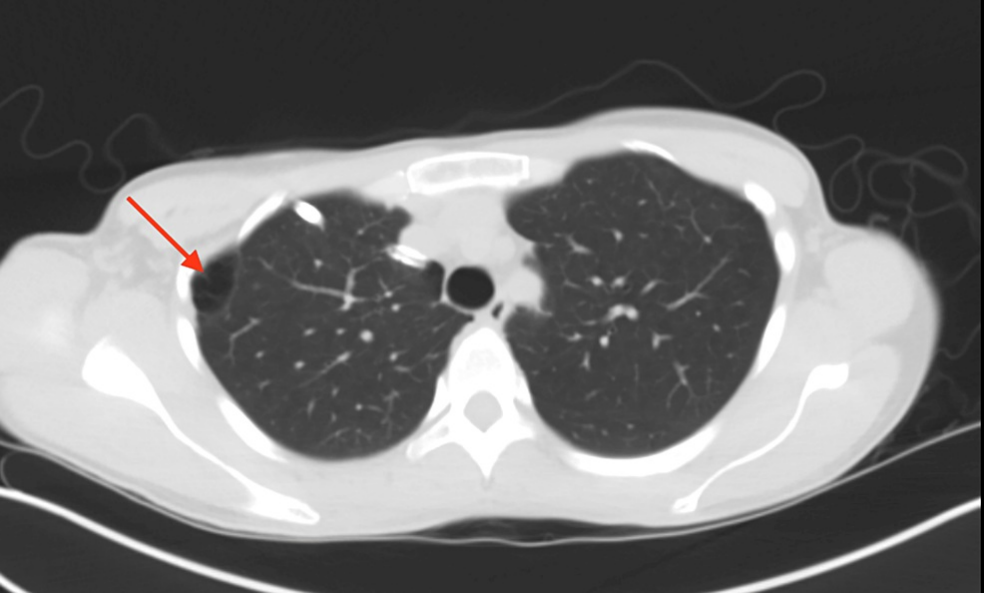
CT Chest revealed a right upper apicolateral bleb.

Thirty days later, she presented to the hospital again with chest pain and shortness of breath. Once again, a right-sided pneumothorax was seen on CXR (Figure 2A), and another thoracostomy tube was placed (Figure 2B). A CT scan revealed a right upper apicolateral bleb (Figure 3). She underwent video-assisted thoracoscopic surgery (VATS) with wedge resection for bleb removal and mechanical pleurodesis. Her thoracostomy tube was eventually removed, and she was discharged in stable condition.

Six months later, the patient presented with intermittent shortness of breath and pain in her right chest that began the previous day. These symptoms had also occurred on two previous occasions since her last outpatient appointment four months earlier. It was noted that these episodes aligned with the cyclic changes of her menstrual cycle. Further inquiry into her gynecologic history revealed a distant diagnosis of pelvic endometriosis. A repeat CXR was ordered to rule out any new complications or etiologies and was negative. She was then referred to her gynecologist for symptomatic care, but did not make her appointment.

**Figure 4:**
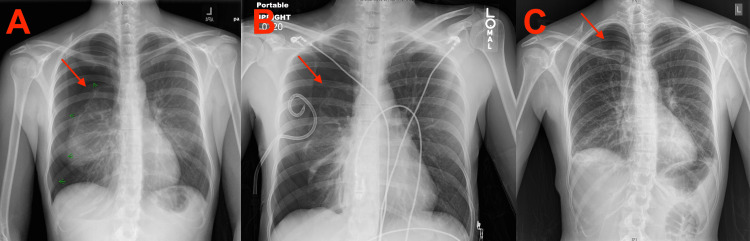
Chest x-ray A showed a third large right-sided recurrent pneumothorax. Chest x-ray B showed a very mild decrease in the right pneumothorax size after thoracostomy tube placement. Chest x-ray C showed a residual small right apical pneumothorax after talc pleurodesis and thoracostomy tube removal.

Two weeks later, at the beginning of her menstrual cycle, the patient again presented to the hospital with worsening right-sided chest pain. A CXR revealed large right-sided pneumothorax (Figure 4A), and another thoracostomy tube was placed (Figure 4B). Subsequently, a repeat thoracoscopy with talc pleurodesis was performed, after which small right apical pneumothorax persisted (Figure 4C). Histologic findings were nonspecific, and no clear evidence of endometriosis was noted. She was started on oral contraceptives and followed up with gynecology. Serial CXRs confirmed decreasing residual pneumothorax over the next year without further relapse.

## Discussion

First reported in 1958, CP has been considered a rare condition [4,6]. However, recent literature suggests that it is the leading cause of SP in women, accounting for nearly a third of all cases [4]. Most cases are right-sided (~90%), and there are no pathognomonic anatomopathological changes [1,5,7]. Despite the advances in medicine, the etiopathology of CP is not fully understood. Though cases of CP are mostly associated with thoracic and pelvic endometriosis, this is not universally documented [1,4]; nevertheless, a detailed past medical and symptom history should be obtained. It is important to note that 20-25% of endometriosis is asymptomatic [8]. If endometriosis is still not suspected, it could suggest that the condition is multifactorial or that the endometriosis causing disease presentation is simply undiscovered at the time of treatment.

There are currently four prevailing hypotheses to explain CP (Table 1).

**Table 1:** Hypotheses to explain the development of catamenial pneumothorax.

Hypothesis	Explanation
Physiologic Hypothesis	High levels of prostaglandin F2 exhibited during menstruation cause bronchospasm and vasoconstriction. This leads to alveolar rupture, especially in the setting of lung bullae or blebs.
Metastatic Hypothesis/Lymphovascular Microembolization Hypothesis	Endometrial cells migrate through blood and lymph vessels and implant at various sites in the body, including the lung parenchyma. The proliferation and necrosis of these endometrial foci during menstruation induce pneumothorax.
Migration Hypothesis	Endometrial tissue migrates preferentially to the right hemidiaphragm due to peritoneal flow, causing diaphragmatic perforations and implantation in and along the right thoracic cavity. The proliferation and necrosis of these endometrial foci during menstruation induce pneumothorax.
Transgenital-Transdiaphragmatic Passage of Air Hypothesis	Lack of cervical mucus during menstruation allows air to pass through the vagina into the uterus, fallopian tubes, and eventually the peritoneal cavity where it migrates into the pleural space through diaphragmatic fenestrations.

Our case most resembles the “physiologic hypothesis," which suggests that vasoconstriction and bronchospasm are caused by high levels of systemic prostaglandin F2 during menstruation, inducing alveolar rupture and pneumothorax [1,4,5]. This can exacerbate underlying conditions such as bullae and/or blebs, and due to its physiologic nature, there is usually an absence of confirmed endometrial lesions in a significant number of these CP patients [5].

The “metastatic” or “lymphovascular microembolization hypothesis" postulates that endometrial cells migrate through blood or lymph vessels to where they implant [1,4,5]. Hemoptysis can be seen with centrally located lesions, while pneumothorax is seen in peripherally located lesions [5]. This theory is supported by findings of endometrial foci in the knee, eye, and brain, with lung parenchyma in some patients [9].

The “migration hypothesis" proposes that pelvic seeding of endometrial tissue migrates preferentially to the right hemidiaphragm due to clockwise peritoneal flow [5]. Catamenial necrosis of diaphragmatic endometrial implants creates perforations, allowing spread into the thoracic cavity. Subsequent implantation on the visceral pleura, followed by catamenial necrosis, leads to pneumothorax [1,5]. This theory is supported by the high prevalence of right-sided thoracic endometriosis and diaphragmatic involvement in CP [5].

Lastly, the “transgenital-transdiaphragmatic passage of air hypothesis" proposes that a lack of cervical mucus during menstruation allows air to pass through the vagina, uterus, fallopian tubes, and eventually into the peritoneal cavity where it can migrate into the pleural space through predominantly right-sided diaphragmatic fenestrations (secondary to congenital defects or endometriosis) [1,5,10]. The theory is supported by cases of CP that have been cured by diaphragmatic plication and tubal ligation [6,7,11]. However, recurrent cases after such interventions suggest this theory does not account for all cases of CP [4].

Treatment of TES consists of both surgical and pharmacological hormonal therapy [1,3-6,9-11]. VATS is considered the gold standard for the diagnosis of thoracic endometriosis [3] and is preferred in the event of pneumothorax [1,4,6]. In addition, thoracotomy involving pleurectomy, bullectomy, or pleurodesis is almost always indicated in the case of recurrent pneumothoraces [1]. Research suggests that the incidence of recurrence is dramatically reduced when pleurodesis is part of the surgical plan [6]. Careful evaluation of diaphragmatic, pericardial, pleural, and pulmonary surfaces should be done to evaluate for endometrial lesions [3,5-6]. According to Slasky et al., diagnostic pneumoperitoneum after peritoneal insufflation has been an effective method for finding occult defects in the diaphragm that could allow the migration of endometrial tissue into the thorax [7]. However, there is contention over which intervention is best to address endometrial lesions within the diaphragm, particularly when no pathology is found. In terms of mechanical intervention, some perform diaphragmatic resection to repair and seal defects, while others use polyglactin or polypropylene mesh [1]. Bagan et al. suggested the placement of a complete diaphragmatic mesh even in the absence of pathology due to the muscle’s high frequency of involvement. They also suggested performing interventions during menstruation to avoid missing hidden lesions [12]. Others prefer combining VATS with video laparoscopy for better visualization of diaphragmatic endometrial lesions. Furthermore, Nezhat et al. suggested that an adjustable-viewing-angle endoscope combined with reverse Trendelenburg positioning and liver retraction should be used during video laparoscopy for the evaluation of pelvic endometriosis to guard against progression to TES [3]. 

Referral to a gynecologist for endometriosis treatment is recommended [1,2,4], as hormonal treatments are an important part of the post-surgical regimen [1,3-6,9-11]. This treatment includes a GnRH analog for 6-12 months (longer in the case of recurrence) to induce amenorrhea for all patients with CP with proven endometriosis [4,10,13]. The intent is to induce hypoestrogenism and a lack of menses, which will atrophy any ectopic endometrium [1]. Suppressing ectopic endometrial activity provides time for pleural adhesions to create effective pleurodesis [3,14]. Other treatments include oral contraceptives and intrauterine devices [1]. Although our patient's hormonal therapy was delayed, Ciriaco et al. showed that estrogen-progesterone pills alone were not an effective post-surgical treatment regimen as the cohort placed on this treatment had a 100% CP recurrence rate [4]. Therefore, a combination and medical and surgical therapy should probably be pursued. However, regardless of treatment modality, the absence of recurrence cannot be guaranteed. Even with surgical intervention, recurrence occurs at a rate of 8-40% at a mean follow-up of four years [1,5], and as in our patient, many will continue to suffer from catamenial chest pain [12].

## Conclusions

It is important to consider CP any time a female of childbearing age presents with SP, particularly in those with a history of endometriosis and recurrent chest pain that correlates with their menstrual cycle. However, it is important not to dismiss the condition simply because of a negative history. An early gynecological referral is crucial, and endometriosis treatment is proven to hinder the progression of TES and reduce the recurrence of CP. 

There are no well-defined guidelines to direct patient care for TES and CP. Further research should be done to determine the most effective treatment plans.
